# Protocol for Work place adjusted Intelligent physical exercise reducing Musculoskeletal pain in Shoulder and neck (VIMS): a cluster randomized controlled trial

**DOI:** 10.1186/1471-2474-11-173

**Published:** 2010-08-05

**Authors:** Lars L Andersen, Mette K Zebis, Mogens T Pedersen, Kirsten K Roessler, Christoffer H Andersen, Mette M Pedersen, Helene Feveile, Ole S Mortensen, Gisela Sjøgaard

**Affiliations:** 1 National Research Centre for the Working Environment, Lersø Parkalle 105, DK 2100 Copenhagen Ø, Denmark; 2 Institute of Exercise and Sport Sciences, University of Copenhagen, Denmark; 3 Department of Occupational and Environmental Medicine, Bispebjerg University Hospital, Bispebjerg Bakke DK 2400 Copenhagen, Denmark; 4 Institute of Sports Science and Clinical Biomechanics, University of Southern Denmark, Odense, Denmark

## Abstract

**Background:**

Neck and shoulder complaints are common among employees in sedentary occupations characterized by intensive computer use. Specific strength training is a promising type of physical exercise for relieving neck and shoulder pain in office workers. However, the optimal combination of frequency and exercise duration, as well as the importance of exercise supervision, is unknown. The VIMS study investigates in a cluster randomized controlled design the effectiveness of different time wise combinations of specific strength training with identical accumulated volume, and the relevance of training supervision for safe and effective training.

**Methods/design:**

A cluster randomized controlled trial of 20 weeks duration where employed office workers are randomized to 1 × 60 min, 3 × 20 min, 9 × 7 min per week of specific strength training with training supervision, to 3 × 20 min per week of specific strength training with a minimal amount of training supervision, or to a reference group without training. A questionnaire will be sent to 2000 employees in jobs characterized by intensive computer work. Employees with cardiovascular disease, trauma, hypertension, or serious chronic disease will be excluded. The main outcome measure is pain in the neck and shoulders at week 20.

**Trial Registration:**

The trial is registered at ClinicalTrials.gov, number NCT01027390.

## Background

Musculoskeletal disorders cause poor work ability and lost working days [1], and constitute a third or more of all registered occupational diseases [2,3]. Among the general population, back and neck pain are the most prevalent types of musculoskeletal disorders, and represent a major socioeconomic burden in terms of sickness absence compensation, disability pension, and health services [4]. Development of neck, shoulder and arm complaints are common in sedentary occupations characterized by intensive computer use [5-7]. Considering the rapid growth of computer-use at all levels of society the problem does not appear to diminish.

Physical exercise is a cornerstone in health and well-being [8]. An increasing number of studies and reviews within the last decade provide evidence for the effectiveness of physical exercise at the workplace in managing musculoskeletal pain [9-12]. While specific strength training of the neck and shoulders is the most promising type of physical exercise for relieving neck pain [13-16], evidence is lacking for the effect of such training on shoulder pain [10]. One study showed effectiveness of a home exercise program in reducing shoulder pain in construction workers [17], but further randomized controlled trials are needed to evaluate the effect of workplace exercise interventions on shoulder, arm and wrist pain [10].

International health guidelines recommend adults to perform at least 30 minutes of moderate physical activity 5 days per week for general health [18]. While these guidelines are based on prevention of metabolic syndrome related disorders, the optimal duration and frequency of physical exercise for proper musculoskeletal function remains to be established. Previous studies on rehabilitation of neck and shoulder pain typically used training frequencies of three times per week with a duration of 20 to 60 minutes per training session [13-16]. However, physiological adaptations in healthy adults can be achieved both in response to long exhausting bouts of resistance training with several days of rest in between and in response to shorter bouts performed several times a week [19]. One study with healthy adults comparing the effect two versus three strength training sessions per week - with equal weekly volume - found similar gains in muscle mass and strength [20]. A meta-analysis - based on 177 resistance training studies - showed that three training days per week is optimal for efficient strength gains in untrained healthy adults without musculoskeletal disorders [21]. By contrast, adherence - at least in weight loss programs - seems to be higher when multiple short bouts of exercise are carried out as opposed to fewer and longer bouts [22]. However, no previous studies have determined the optimal combination of duration and frequency of exercise for relieving pain of the neck and shoulder. Resolving these research questions are important, to thereby allow companies a more flexible and efficient integration of exercise at the workplace.

Most studies investigating the effect of exercise for neck and shoulder pain have used supervision by experienced training instructors or physiotherapists [13-16]. However, this may in practice not be an available resource at most workplaces. Studies investigating the effect of unsupervised training at the workplace on musculoskeletal pain have typically reported small or insignificant treatment effects [10]. Thus it is necessary to determine the minimal amount of supervision needed to effectively implement exercise at the workplace.

This study - Work place Adjusted Intelligent physical exercise reducing Musculoskeletal pain in Shoulder and neck (VIMS) - investigates in a cluster randomized controlled design

1) the effect of different time wise combinations of training with identical accumulated duration, in order to elucidate possible ranges of training effectiveness and flexibility

2) the relevance of training supervision for safe and effective training, in order to minimize expenses for workplace physical exercise training.

The concept of 'intelligent physical exercise' is to balance the physiological capacity relatively to occupational exposure, tailor the exercise to individual capacities and disorders, allow for flexibility and interest for the participant, and to be as cost-effective for the company as possible.

## Methods and design

### Study design

We are currently conducting a cluster randomized controlled trial in Denmark. The trial began in February 2010 and ends in June 2010. The participants were recruited from 12 geographically different units located in all major cities throughout Denmark balanced according to the population density with around half in the Copenhagen area and half in other parts of Denmark. The criteria were that the sites should be distributed across the country, they should be so large that it was possible to randomize naturally occurring clusters to five groups and the total number of employees in the sites should be around 2000 persons.

All of the participants gave their written consent to participate in the study. The local ethics committee approved the study protocol (H-C-2008-103), which qualified for registration in the ClinicalTrials.gov, number NCT01027390

### Study population

**Figure 1:**
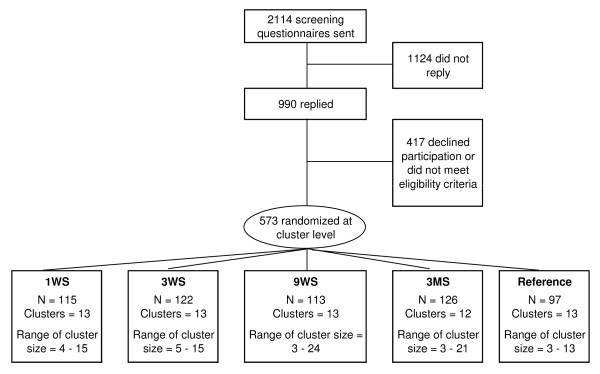
**Flow-chart**. 1WS: one weekly session with supervision, 3WS: 3 weekly sessions with supervision, 9WS: 9 weekly sessions with supervision, 3MS: 3 weekly sessions with minimal supervision. Reference: reference group without training

Figure 1 shows the flow of participants. We invited 2114 employees to participate in the study. A short introduction and invitation text, together with a link to an internet-based questionnaire on current working conditions, health and symptoms, physical activity etc. (for details see below) went out to the prospective participants by email. Out of the invited employees, 990 replied to the questionnaire. Table 1 shows that employees (men and women separately) who accepted (yes) and declined (no) participation were broadly similar with regard to gender, age, height, and body mass. However, employees accepting participation had a higher 12-month prevalence of musculoskeletal symptoms in the neck and shoulders, and higher pain intensity in the neck and shoulders during the previous 3 months.

**Table 1:** Characteristics of employees who accepted (yes) and declined (no) participation in the intervention.

	Women (N = 570)			Men (N = 420)		
	Yes	No	p - value	Yes	No	p - value
Age (years)	44 (11)	42 (12)	0.04	49 (9)	48 (11)	0.19
Height (cm)	168 (6)	170 (6)	0.01	184 (6)	183 (6)	0.61
Weight (kg)	70 (13)	69 (12)	0.62	90 (13)	88 (11)	0.42
BMI (kg m^-2^)	25 (4)	24 (4)	0.09	27 (3)	26 (3)	0.47
Neck pain intensity previous 3 months (scale 0-9)	3.8 (2.3)	2.5 (2.4)	<.0001	2.7 (2.3)	1.7 (2.0)	<.0001
Shoulder (right) pain intensity previous 3 months (scale 0-9)	2.6 (2.5)	1.6 (2.1)	<.0001	1.9 (2.3)	0.8 (1.5)	<.0001
Shoulder (left) pain intensity previous 3 months (scale 0-9)	2.1 (2.4)	1.3 (2.0)	<.0001	1.4 (2.1)	0.6 (1.4)	<.0001
12-month prevalence of neck pain	94%	78%	<.0001	85%	63%	<.0001
12-month prevalence of right shoulder pain	73%	53%	<.0001	59%	35%	<.0001
12-month prevalence of left shoulder pain	64%	41%	<.0001	49%	26%	<.0001

P-values denote significance levels between those accepting and declining participation, separately for women and men.

Exclusion criteria were (i) hypertension (Systolic BP >160, diastolic BP > 100) or cardiovascular diseases, (ii) symptomatic herniated disc or severe disorders of the cervical spine, (iii) postoperative conditions in the neck and shoulder region, (iv) history of severe trauma, and (v) pregnancy, (vi) or other serious disease. Prospective participants had to work at least half of their working hours in an office environment to be included. The remaining 573 participants were randomly allocated at the cluster level to five groups as described below. Table 2 shows the baseline characteristics of the included participants of the five groups. After the cluster randomization, workers were excluded if they contracted a condition included in the aforementioned exclusion criteria or if they were on maternity leave or on other long-term absences (men and women).

**Table 2:** Characteristics of the five groups.

	1WS (N = 115)	3WS (N = 122)	9WS (N = 113)	3MS (N = 126)	Reference (N = 97)
Age (years)	47 (11)	47 (11)	46 (10)	45 (11)	46 (10)
Height (cm)	174 (9)	174 (10)	175 (9)	175 (10)	175 (10)
Weight (kg)	77 (15)	76 (18)	77(14)	78 (16)	80 (16)
BMI (kg^.^m^-2^)	25 (4)	25 (4)	25 (4)	26 (4)	26 (5)
Neck pain intensity previous 3 months (scale 0-9)	3.3 (2.2)	3.2 (2.4)	3.1 (2.3)	3.3 (2.3)	3.2 (2.3)
Shoulder (right) pain intensity previous 3 months (scale 0-9)	2.2 (2.3)	2.3 (2.4)	1.9 (2.2)	2.0 (2.4)	2.0 (2.3)
Shoulder (left) pain intensity previous 3 months (scale 0-9)	1.5 (2.1)	1.7 (2.2)	1.8 (2.2)	1.6 (2.2)	1.4 (1.8)
12-month prevalence of neck pain	89%	89%	88%	92%	90%
12-month prevalence of right shoulder pain	69%	69%	63%	66%	62%
12-month prevalence of left shoulder pain	54%	58%	59%	56%	53%

1WS: one weekly session with supervision, 3WS: 3 weekly sessions with supervision, 9WS: 9 weekly sessions with supervision, 3MS: 3 weekly sessions with minimal supervision. Reference: Reference group without training

### Procedure of the cluster randomization

Work place physical exercise has implicit social elements of both getting together, competition and group pressure. For the intervention to capture these elements, we used cluster as the unit of randomization. The clusters were naturally occurring groups of employees, thereby also minimizing contamination between clusters. To help ensure the comparability of the 4 intervention groups and the reference group, geographical sites were categorized into 13 strata. Each stratum was divided into 5 naturally occurring clusters. Building, floor, department and the size of clusters were taken into account when defining the clusters in order to attain intervention and reference groups of similar sizes in each stratum. Strata and clusters were formed by authors MZ, LA and GS. In two of the strata forming of clusters entailed individual randomization into two clusters. This individual randomization was performed by a data manger when stratum and cluster affiliation was attached to the participants.

Within each stratum the clusters were randomized to group (group was sampled with replacement and assigned to the cluster). Randomization was performed by author HF using a SAS macro based on the RANUNI function. In more details: Strata were numbered consecutively (1,...,13) and clusters numbered consecutively with strata (1,...,5). Within each stratum, the clusters were sorted according to a random number and assigned the color codes red, green, blue yellow white (a code for the 4 interventions and the reference group) in succession and cyclically. To minimize imbalance over several strata with a number of clusters no divisible by five, the SAS macro was programmed to start the cyclic assignment by assigning one of the colors at random (with probability 1/5). As the formation of clusters resulted in exactly five clusters in each stratum, this element of the macro was redundant.

### Sequel to the randomization

When the training schedules and premises were sent out, just before the start of the intervention, it appeared from the enquiries that a particular subgroup of participants had misunderstood the slightly ambiguous question concerning geographical site. The wrongly placed 43 individuals were identified by comparing questionnaire responses to staff records. Before the intervention commenced the individuals were re-allocated according to the following principles wherever possible:

▪ From the response to questions concerning departmental affiliation and floor of the building the true cluster was determined. The respondent was re-allocated to the treatment corresponding to that cluster.

▪ When ambiguity with respect to determining the right re-allocation cluster, a cluster allocation resulting in no change in treatment group was preferred.

▪ If the respondent belonged in one of the clusters detemined by individual randomisation, the cluster re-allocation was determined by flip of a coin.

### The VIMS intervention program

The four training-groups performed the same total amount of exercises and repetitions per week - i.e. an equal training volume - for a total of one hour per week for 20 weeks during working-hours. Experienced instructors supervised half of the training sessions three of the groups. The first group trained for 1 hour once a week (1WS, 1 per week, supervised), the second group trained 20 minutes 3 times a week (3WS, 3 per week, supervised) and the third group trained 7 minutes 9 times a week (9WS, 9 per week, supervised). Group four (3MS, 3 per week, minimally supervised) followed the same program as 3MS, but received supervision only during the initial week. The reference group was not offered any physical training, but replied to the same questionnaires as the intervention-groups.

**Figure 2:**
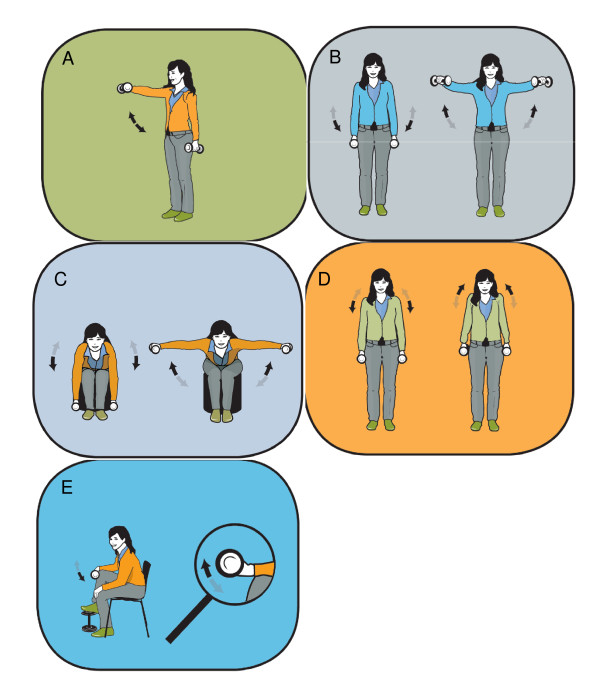
**Illustration of the five training exercises**; A) Front raise, B) lateral raise, C) reverse flies, D) shrugs, and E) wrist extension.

The intervention-groups performed specific strength training with 5 different dumbbell exercises, as shown in figure 2, for the neck and shoulder muscles:

1. Front raise: From a neutral starting position the participant lifts one arm at a time to 90 degrees shoulder flexion, and 90 degrees internal rotation. The elbows are slightly flexed (~5°) during the entire range of motion.

2. Lateral raise: the participant is standing with arms in neutral starting position and the elbows are in a static slightly flexed position (~5°). The participant lifts both arms to 90 degrees shoulder abduction and 30 degrees horizontal flexion.

3. Reverse flies: The participant is sitting bent over forward with the back straight and arms hanging. The arms are raised bilaterally, while keeping the elbows in a static slightly flexed position (~5°), until the upper arms are horizontal.

4. Shrugs: The participant is standing erect with arms to the side and elevates the shoulders as high as possible in a maximal shrug.

5. Wrist extension: sitting with the forearm pronated on a support. From full palmar flexion the participant moves the wrist to full dorsal flexion

Participants performed exercises in a rotating manner to optimally increase training load, and rested 1-2 minutes between sets [19]. Each training session started by warming up for 10 repetitions with loadings of 50% of 1 repetitions maximum for each respective exercise of that day.

During the 20-week intervention training loads were progressively increased according to the principle of periodization and progressive overload. Table 3 shows that the training intensity was progressively increased from 20 repetitions maximum (RM) at the beginning of the intervention-period to 8 RM during the later phase. The first 12 weeks of the program followed the principles of linear periodization and the last 8 weeks the principles of undulating periodization, as both methods have been shown to be more efficient than non-periodized training [19].

**Table 3:** Progression schedule

Week	Sets and intensities
1	15 × 20 RM
2	15 × 15 RM
3	15 × 15 RM
4	21 × 15 RM
5	21 × 12 RM
6	21 × 12 RM
7	21 × 12 RM
8	24 × 12 RM
9	21 × 10 RM
10	21 × 10 RM
11	24 × 10 RM
12	24 × 10 RM
13	6 × 8 RM, 6 × 12 RM, 6 × 15 RM
14	9 × 8 RM, 6 × 12 RM, 6 × 15 RM
15	9 × 8 RM, 6 × 12 RM, 6 × 15 RM
16	21 × 8 RM
17	9 × 8 RM, 9 × 12 RM, 6 × 15 RM
18	9 × 8 RM, 9 × 12 RM, 6 × 15 RM
19	9 × 8 RM, 9 × 12 RM, 6 × 15 RM
20	24 × 8 RM

Total number of sets per week and intensities used during the 20 week intervention period. All four training groups had the same weekly training volume. RM: repetitions maximum. For instance 15 × 20 RM should be read "15 sets with loadings of 20 repetitions maximum".

#### Exercise adjustments in case of pain

If a participant experienced incidence of joint pain or the like during a specific exercise, we asked them to adjust the exercise as follows: First, the instructor asked the participant to externally rotate the arms, slightly alter the path of the arms or reduce the range of movement during the exercise. Then, the participant reduced the training load of the exercise (e.g. a reduction of 1 kg). If this did not help, the participant reduced the number of sets, and finally reduced training frequency. If none of these adjustments had the desired effect, the exercise was removed from the participant's training program for at least 1 week.

#### Motivation

Compliance to training is challenging. Workplace interventions have typically reported low to moderate compliance [23,24]. In the VIMS-study, we focused especially on the environment. We tried to improve compliance by placing the training facilities close to working offices to reduce transportation time and distance as a barrier for not training. Whenever possible, we chose bright rooms and made efforts to make the rooms appealing. We ensured sufficient space to move freely. Colored posters illustrating the exercises with instructions were hung on the walls. The participants received a training diary for registering training sessions and loads. We encouraged participants to train during a specified time period together with their colleagues. Instructors supervised half of the training sessions in groups 1WS, 3WS and 9WS. Instructors taught the participants how to perform the exercises, and helped with exercise adjustment when needed. The instructors focused on positive feedback to maintain motivation. The instructors met regularly to discuss positive and negative experiences and possible problems. A mail-support was created for the participants in case of questions or problems regarding the exercises.

### Self-reported measures

We applied a structured email-based questionnaire including e.g. the Standardized Nordic questionnaire for musculoskeletal disorders [25], the International Physical Activity Questionnaire (IPAQ) [26], self-efficacy [27,28], stages of change [28-32], and work productivity [33]. The main questions are described in more detail below.

*Musculoskeletal pain symptoms *of the neck, shoulder, arm, hand, and back were evaluated using validated scales concerning both intensity and duration of symptoms. Participants replied to the questions "How many days have you had trouble in [body part] during the last three months?" (0 days; 1-7 days; 8-30 days; >30 days; everyday) for symptom duration, and "On average, how intense was your pain in [body part] during the last three months on a 0-9 scale?" (where 0 means no complaints and 9 means pain as bad as it can be) for symptom intensity. Answers to the question that concerned symptom duration were recoded as follows: 0 days = 0, 1-7 days = 4, 8-30 days = 19, >30 days = 60, everyday = 90. Illustrations from the Nordic questionnaire defined the respective body regions [25]. Further, headache was evaluated using a questionnaire on intensity, duration, and frequency of headache during the previous month.

*The level of physical activity *were assessed using the International Physical Activity Questionnaire (IPAQ) [26]. Total physical activity and vigorous-intensity activity during work, transportation, housework or gardening, and leisure were converted to metabolic equivalent task (MET minutes per week) according to the guidelines for data processing of the IPAQ. Also, participants were classified into the High, Moderate, or Low category based on their level of activity. Participants in High performed on a weekly basis: a) vigorous-intensity activity on at least 3 days, achieving a minimum of 1500 MET minutes per week; or b) different activities 5 or more days achieving a minimum of 3000 MET minutes per week. Participants in Moderate performed: a) 3 or more days of per week of vigorous-intensity activity of at least 20 min per day; or b) 5 or more days per week of moderate-intensity activity or walking of at least 30 minutes per day; or c) 5 or more days of different activities achieving a minimum of 600 MET minutes per week. Participants who did not meet the criteria for the two other categories were placed in the Low category.

*Self-efficacy *in relation to barriers towards physical activity was assessed by a questionnaire [27,28]. E.g. "I feel convinced that I am able to exercise 3 times or more a week with a duration of at least 20 minutes at a time even though: "I am under a lot of stress", "I feel I don't have the time", "I have to exercise alone", "I don't have access to exercise equipment", "I am spending time with friends or family who do not exercise", and "It's raining or snowing".

*Stages of change *in relation to physical activity was in this study assessed by a questionnaire originally presented by Marcus and colleagues in 1992 [27] but further developed by Benisovich in 1998 [28-32]. Questions asked in the questionnaire were e.g. "As far as I'm concerned, I don't need to exercise regularly", "I really think I should work on getting started with a regular exercise program in the next 6 months", and "I have started exercising regularly within the last 6 months".

*Productivity *was rated on an 11-step ordinal scale: "How do you perceive your overall productivity the last 4 weeks?" The rating went from 0 (the worst a worker could do) to 10 (the best a worker in the same job could do) [33]

### Objective measures

Testing of physical capacity was performed at the beginning, midways, and at the end of the 20-week intervention in all five groups.

*Maximal muscle strength *was assessed by a repetition maximum (RM) test at baseline, at midterm (10 weeks) and at the end of the intervention (20 weeks). We used the bilateral lateral raise exercise (i.e. from neutral position to 90 abduction/30 horizontal flexion). The participant stood with the back against a wall, and performed 1 repetition with 1 kg dumbbells and held the top-position for 1-2 seconds, waited 30 seconds, and then one repetition with 2 kg dumbbells. Using this procedure, the examiner added 1 kg until the participant was unable to lift and hold the dumbbell properly. The maximal weight lifted with acceptable technique was noted as 1 RM.

*Strength-endurance *was assessed with a submaximal load. Using the same exercise as described above, the participant performed as many repetitions as possible with a load of 1 kg less than the 1 RM load. During each repetition the participant held the top-position for 1 second. The participant used the same load during midterm and end of intervention as the one used during the baseline test.

### Statistics

**Figure 3:**
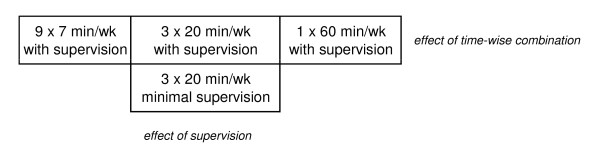
**Schematic illustration of the four training groups**. Using this design we can compare the effectiveness of both time-wise combinations of training and the influence of training supervision.

The primary outcome is change in pain of the neck and shoulders at 20 weeks. Secondary outcomes include the other measures mentioned above. We plan to analyze the main data according to the intention-to-treat principle. Secondly, we will also analyze data per protocol. We will use repeated measures analysis of variance. Our design allows us to compare both the effect of different combination of duration and frequency and the effect of training supervision (figure 3). The following null-hypotheses will be tested;

1) There is no difference between the groups 1WS, 3WS, 9WS and reference for the change in neck/shoulder pain from baseline to week 20.

2) There is no difference between the groups 3WS, 3MS and reference for the change in neck/shoulder pain from baseline to week 20.

Power analyses performed prior to the study showed that - to reject the null-hypothesis of equality - we should include 150 participants per group (allowing for a 20% loss to follow-up) for 80% power to detect a 10% change in pain between groups.

## Competing interests

The authors declare that they have no competing interests.

## Authors' contributions

GS, MZ, OM, LA and MTP were responsible for the research design. LA drafted the paper, and all co-authors made significant contributions to drafting the protocol. All authors have read and approved the final manuscript.

## Pre-publication history

The pre-publication history for this paper can be accessed here:

http://www.biomedcentral.com/1471-2474/11/173/prepub
